# Use of Homecare Nursing and Sense of Security Among Family Caregivers of Older Adults

**DOI:** 10.1093/geroni/igaa057.501

**Published:** 2020-12-16

**Authors:** Ayumi Igarashi, Madoka Katayama, Mariko Sakka, Maiko Noguchi-Watanabe, Chie Fukui, Asa Inagaki, Satomi Kitamura, Noriko Yamamoto-Mitani

**Affiliations:** The University of Tokyo, Tokyo, Japan

## Abstract

Having a sense of security for living at home is essential to continue living at home and it is important to develop a community care system that enables high sense of security. Because frail to dependent older adults often accompany various conditions that require medical attention, receiving homecare nursing might allow the family caregiver to feel safe in living at home. Therefore we examined whether receiving homecare nursing service contributes to a higher family caregiver sense of security. We conducted a questionnaire survey regarding older adults who were 75 years and older with at least some sort of community care services, and their family caregivers (n=776). Family sense of security was measured using 5 items (e.g., “I feel secure about my relative living at home with help of available services”), rated on 5-point Likert scales. Characteristics of older adults were obtained from their nurses or care managers. The mean age of the older adults was 85.8 years, 58% female, and 8 % living alone. The mean total family sense of security was 20.6 (standard deviation = 3.7) out of 25. In multiple regression analysis, the family sense of security was positively associated with the use of homecare nursing (β= 0.09, p=0.020), adjusting for participants’ age, stability of their medical conditions, level of activities of daily living, use of medical procedures, living arrangements, and house call physician services. Homecare provided by nurses could contribute to longer staying at home among older adults by way of the higher family sense of security.

